# Unveiling the presence of ESBL-producing coliform bacteria in the aquaculture system of Cumilla District of Bangladesh

**DOI:** 10.1016/j.nmni.2026.101706

**Published:** 2026-01-16

**Authors:** Rakibul Islam, Nazia Afrin, Md Shakhawate Hossain

**Affiliations:** a Department of Fisheries Biology and Aquatic Environment, Gazipur Agricultural University, Gazipur, 1706, Bangladesh; b Laboratory of Microbiology, Department of Botany, Jahangirnagar University, Savar, 1342, Bangladesh

**Keywords:** Aquaculture water, Antibiotic resistance, ESBL-Producing Bacteria, Coliforms, Multidrug resistance (MDR), qPCR

## Abstract

The increasing use of antibiotics in aquaculture has raised global concerns about the emergence and spread of antimicrobial resistance (AMR) in aquatic environments.

**Objectives:**

This study investigated the presence of extended-spectrum β-lactamase (ESBL) producing coliform bacteria and their antibiotic resistance profiles in aquaculture systems across six Upazilas of the Cumilla district of Bangladesh.

**Methods:**

Water quality parameters (pH, dissolved oxygen, temperature, and ammonia) were measured with HACH kits. Bacterial isolates were screening through culture dependent methods and biochemical tests. Antibiotic susceptibilities were tested by disc diffusion method and resistant genes were identified using qPCR. Isolates ESBL were confirmed by double disc synergy test (DDST).

**Results:**

Microbiological analysis revealed significant variation in the bacterial contamination across sites. The most dominant bacteria were *Escherichia coli*, *Klebsiella* spp., *Enterobacter* spp., and *Salmonella* spp. All isolates were resistant to multiple antibiotics and multi-drug resistance index (MDRI) values exceeded the threshold 0.2. Isolates showed higher resistance against β-lactam antibiotics. Phenotypic ESBL testing identified 34.3 % of isolates as ESBL producers. The qPCR analysis confirmed the presence of multiple antibiotic resistance genes (ARGs), including bla SHV, bla TEM, bla CMY, bla CTX-M15, Sul1, Sul2, and tetracycline resistance genes, along with mobile genetic elements (MGEs) such as TSO.

**Conclusion:**

The findings suggest that aquaculture environments in the Cumilla District may serve as a significant reservoir of multi-drug resistant and ESBL-producing coliform bacteria. Responsible antibiotic practices and regular surveillance are necessary to limit the spread of AMR in the aquaculture industry of Bangladesh.

## Highlights

• Prophylactic use of antibiotic in aquaculture is a global concern to surge AMR.
• ESBL- producing coliform bacteria have been identified in aquaculture waters.
• Dominant bacteria were *Escherichia coli*, *Klebsiella* spp., *Enterobacter* spp., and *Salmonella* spp.
• Multi-drug resistance index (MDRI) was greater than the threshold value 0.2.
• The presence of antibiotic resistance genes (ARGs) was confirmed in isolated bacteria.

## 1 Introduction

Aquaculture, the farming of aquatic organisms, has emerged as a significant contributor to global food production, catering to a substantial portion of the world's seafood consumption [1]. In Bangladesh, freshwater aquaculture plays a pivotal role in meeting the country's protein needs, particularly in regions like Cumilla, where small-scale carp aquaculture systems predominate [2]. The aquaculture sector in Bangladesh has witnessed steady growth over the years, with inland capture fisheries and aquaculture making significant contributions to the nation's economy and food security [3]. Aquaculture's economic significance extends beyond food production, encompassing job creation, food security enhancement, foreign income generation, and socioeconomic advancement. In Bangladesh, the fishing industry substantially contributes to the national economy as it accounts for about 2.53 % of GDP and 11.55 % of agricultural GDP in the financial year 2023–2024 [4]. Globally, aquaculture production reached 94.4 million tons by weight and US$164.6 billion in value in 2022, underscoring its importance in meeting the growing demand for aquatic products [5]. These are the results of intensification of aquaculture system.

The use of aqua medicines in aquaculture systems (semi-intensive/intensive), especially antibiotics, meets various purposes, like disease prevention and treatment. Generally, antibiotics like Amoxicillin, Oxytetracycline and Sulphadiazine-trimethoprim are used to treat bacteria and bacterial infections [6]. Studies have revealed that non-selective use of antibiotics in aquaculture promotes the evolution and dispersal of antibiotic resistance genes (ARGs) in aquatic environments, with antibiotic-resistant coliform bacteria causing significant threats to public health [7]; [8]. The transfer of resistance genes from aquatic bacteria to human pathogens through horizontal gene transfer mechanisms further exacerbates the threat posed by antibiotic-resistant bacteria [9].

The global widespread occurrence of antimicrobial resistance (AMR) is a crucial public health challenge if it is left unaddressed, with forecasts of over 10 million deaths yearly by 2050 (Chinedelevitch et al., 2022). The World Health Organization (WHO) identified for example, *Klebsiella pneumoniae*, *Escherichia coli*, *Enterococcus* spp., and *Staphylococcus aureus* as priority antibiotic-resistant bacteria (ARB) most concerning pathogen [10]. In Bangladesh, escalating human consumption and indiscriminate use of antibiotics (prescribed and non-prescribed) in aquaculture system and detection of multi-drug-resistant bacteria underscore the immediate need for interventions to battle antimicrobial resistance [11].

ESBL-producing bacteria such as coliform (*Escheriachia coli, Klebsiella pneumoniae*) are capable of hydrolyzing extended-spectrum beta-lactam antibiotics, making them ineffective in treating disease caused by these bacteria [12]. The misuse and overuse of antibiotics in clinical settings and aquaculture operation facilities the spread of ESBL producing bacteria [13,14]. The aquatic environments are ideal environments for the selection and dissemination of these antimicrobial resistant ESBL- producing bacteria through the collection and discharge of effluents having antibiotic residue and ABR bacteria into surrounding environments [15].

The Cumilla District of Bangladesh is a significant center for aquaculture activities in the east-south part of Bangladesh. However, there is scant information available regarding the prevalence of ESBL-producing coliform bacteria in the aquaculture settings in this region. Understanding the occurrence and dispersal of ESBL producing coliform bacteria is vital for generating effective approaches to alleviate its spread and lessen its effects on human health and environment [16]. Therefore, this study was intended to assess the occurrence of antibiotic -resistant ESBL producing coliform in the aquaculture ponds of the Cumilla District and identify their associated risk factors.

## 2 Materials and methods

### 2.1 Study site selection, collection of samples and processing

**Fig. 1:**
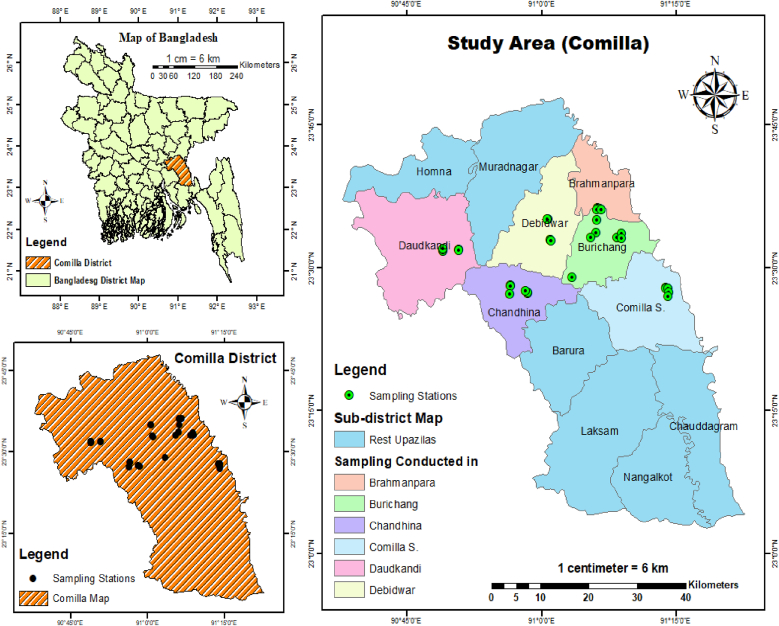
Map showing the sample collection sites of Cumilla district.

Water samples were collected from 6 different upazilas of Cumilla District which included Comilla Adarsha Sadar, Burichong, Brahmanpara, Chandina, Debidwar, and Daudkandi upazila. For each upazila, samples were collected from 6 different locations (Fig. 1, Table S1). Samples were collected in sterile 500 ml plastic bottle and preserved in ice box until reach to laboratory. After reaching the laboratory, samples were stored at 4 °C until further analysis.

### 2.2 Water quality measurements

Aquaculture necessitates monitoring various critical abiotic parameters to comprehend aquatic conditions effectively like temperature, turbidity, salinity, dissolved oxygen (DO), carbon dioxide (CO_2_), total ammonia (NH_3_), pH, and water hardness. Measurement was taken on dissolved oxygen (DO), temperature, pH by using digital meter in the sampling spot and ammonia (NH_3_) was tested by using colorimetric method in the laboratory.

### 2.3 Isolation of bacteria and determination of microbial load

For determination of microbial load in water samples, serial dilutions of samples were made up to 10^−7^ with sterile normal saline. 0.1 ml of each dilution was evenly spread on the plate count agar (PCA) medium and incubated at 37 °C for 24 h. Plates were screened for the presence of discrete colonies after incubation period and the actual numbers of bacteria were estimated as colony forming unit per ml (CFU/ml). Colony forming unit (CFU/g) was calculated using the following equation - CFU/ml = Number of colonies on the agar plates/(dilution factor of the tube × amount plated) [17].

### 2.4 Calculation of total coliform count

Violet red bile agar (VRBA), which is a selective and differential medium commonly used for the detection and enumeration of coliform bacteria, particularly *Escherichia coli* (*E. coli*), in water and food samples. After incubation, plates are examined for the presence of pink to red colonies which are presumptive coliforms. The number of colonies is counted, and the results are reported as colony-forming units (CFU) per unit volume of sample. For total coliform count the same procedure of total viable plate count is followed and total coliform count is calculated using the same equation.

### 2.5 Identification of bacterial isolates through selective and differential media culture and biochemical tests

Bacterial isolates were grown on EMB and MacConkey agar media for identification of coliforms and IMViC (Indole, Methyl red, Voges-Proskauer and Citrate test) tests were used for further identification of coliform bacteria.

### 2.6 Pure culture of selected bacterial isolates and glycerol stocking

Pure culture of bacterial colony was done using spread plate method. After finding the pure colony, these bacterial isolates were stocked in glycerol as method described in Khatun et al. [18].

### 2.7 Antibiotic susceptibility testing and multi-drug resistance index calculation

An antibiotic sensitivity test was carried out for selected strains on the most common antibiotics by disc diffusion technique. About 10 μl of the 4 h sub-culture broth with an Optical Density value ranging from 0.8 to 1.0 of the strain was spread on the Mueller-Hinton agar media and, antibiotic Bio-discs were subsequently placed on plates. Finally, the plates were incubated at 35 °C–37 °C for 24 h to observe and measure the inhibition zone as method describe in Khatun et al. [18] according to European Committee on Antimicrobial Susceptibility Testing [19]. The interpretations and zone sizes were illustrated based on a table of the following antibiotic susceptibility testing breakpoints table (Table S2).

### 2.8 Calculation of multi-drug resistance index

The multi-drug resistance index (MDRI) for each bacterial isolate against the antibiotics tested were calculated using a formula. MDRI is calculated by the ratio of number of resistant antibiotics to which isolate is resistance to total number of antibiotics to which organism is tested [20].

$$MDRI=\frac{a}{b}$$

Where, a = Number of antibiotics to which the bacteria is resistant.

b = total number of antibiotics tested to bacteria.

### 2.9 Detection of ESBL producing bacteria

**Fig. 2:**
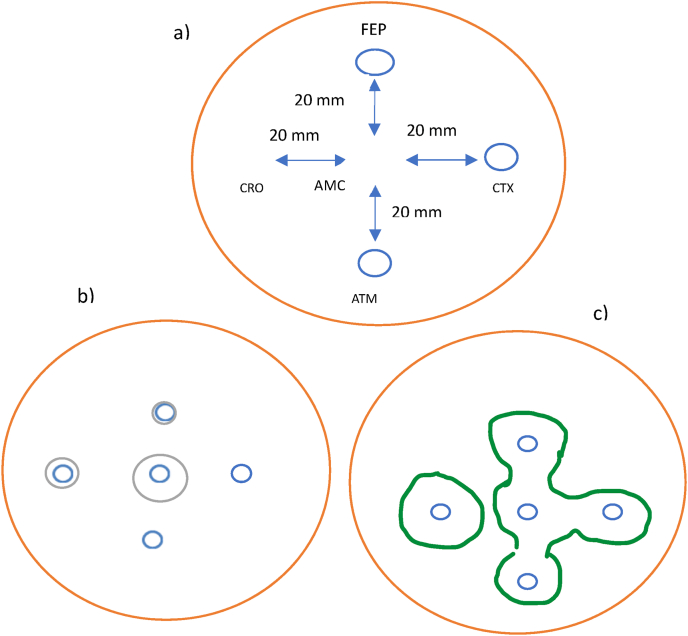
(a) Disc placement of double disc synergy test (DDST) for identifying ESBL positive isolates; (b) ESBL negative isolate and (c) ESBL positive isolate.

Each isolate was processed through double disc synergy test (DDST). DDST was performed using five antibiotic discs (OXOID); Amoxicillin + Clavulanic acid (AMC 10 μg), Aztreonam (ATM 30 μg), Ceftriaxone (CRO 30 μg), Cefepime (FEP 30 μg) and Cefotaxime (CTX 30 μg). AMC (10 μg) was placed at the center of Mueller–Hinton agar plate containing inoculum of desired isolate. Other discs were placed 20 mm apart from AMC [21]. Plate-containing discs was placed inverted in incubator at 37 °C overnight. The plate was observed for extension of inhibitory zone from 3rd generation cephalosporins toward AMC forming a characteristic shape denoted as “keyhole” is indicative as ESBL production (Fig. 2).

### 2.10 Antibiotic resistance genes in selected isolates through RT-PCR analysis

#### 2.10.1 Total RNA extraction

Total RNA was extracted from selected bacterial isolates using spin or vacuum (SV) total RNA extraction kit (Promega, USA) following the manufacturer's instructions. Total RNA concentration and purity were determined using a NanoDrop 2000 spectrophotometer (NanoDrop, Thermo Scientific, USA).

#### 2.10.2 cDNA synthesis

Before cDNA synthesis, total RNA was treated with amplification grade DNase I (Promega, USA) to remove any traces of genomic DNA according to the manufacturer's instructions. Subsequently, the first strand complementary DNA (cDNA) was synthesized from 2 μg of total RNA using GoScriptTM Reverse Transcription System (Promega, USA) according to the manufacturer's instructions.

#### 2.10.3 Quantitative real time PCR (qPCR) analysis of reference genes

Genes determined in the study were-lactamases group, sulfonamide group and tetracycline resistance genes Group. A pure colony selected isolate with template cDNA was added into the reaction mix composed of 1 L of each primer (except tet genes, where was the volume of primers 0.5 L) and DNAfree PCRwater in a total volume of 25 L. Primers used during each PCR are listed in Table S3. All reactions were carried out according to the supplier's instructions. The cycling parameters used were as follows: an initial denaturation (2 min at 95 °C), followed by 40 cycles of 10 s at 95 °C, 30 s at optimized temperatures for specific genes, and 30 s at 72 °C with fluorescence measured at the end of the annealing and extension steps. At the end of each qRT-PCR reaction, PCR products were subjected to a melt curve analysis to confirm the presence of a single amplicon. Melting curve analysis was performed by increasing the temperature from 65 °C to 95 °C at a rate of 0.2 °C/sec with continuous fluorescence at every 0.5 C increments. All samples were run in triplicate. The comparative Cq (ΔCq) method was used to calculate the changes in gene expression as a relative-fold difference between the control and treated sample. All the procedures of real time PCR were performed following MIQE guidelines.

#### 2.10.4 Statistical analysis

Data were presented as mean ± SD (standard deviation). One way analysis of variance followed by Duncan's multiple range test was conducted at 5 % level of significance with the help of statistical software R 4.4.2 and Microsoft Excel.

## 3 Results and discussion

### 3.1 Water quality parameters

Water quality parameters such as pH, dissolved oxygen, temperature, and ammonia play a critical role in sustaining healthy aquaculture environments [22]. When we compare the water quality parameters upazila wise, we found notable variations in pH, dissolved oxygen (DO), temperature, and ammonia concentrations (Table S4). The observed pH values across most districts remained within the optimal range of 6.5–8.5 recommended for aquaculture [23], though the elevated pH in Comilla Adarsha Sadar (8–10.0) could affect nutrient availability and fish metabolism [22]. Dissolved oxygen levels below 5 mg/L, as found in Daudkandi and Brahmanpara (2.1 and 3.2 respectively) (Fig. 3), may cause stress to fish and reduce growth rates [23,24]. The high DO in Comilla Adarsha Sadar (4.5–10.2 mg/L) might reflect photosynthetic activity but can also lead to oxygen supersaturation and associated physiological stress [25]. Temperature ranges across most districts were generally within acceptable limits for tropical aquaculture (26–32 °C) [26], but the elevated readings in Burichong (above 34 °C) may induce thermal stress during hotter months. Total ammonia levels, though mostly low, exceeded recommended limits (<0.05 mg/L for unionized ammonia) in Burichong and Comilla Adarsha Sadar, potentially causing toxicity and stress in fish. Poor water quality parameters in aquaculture pond can exacerbate bacterial proliferation and resistance development, influencing both aquatic health and public health outcomes associated with aquaculture systems [27]; [28].

Fig. 1. Water quality parameters of aquaculture water collected from Cumilla Districts.

### 3.2 Total plate count and total coliform count

**Fig. 3:**
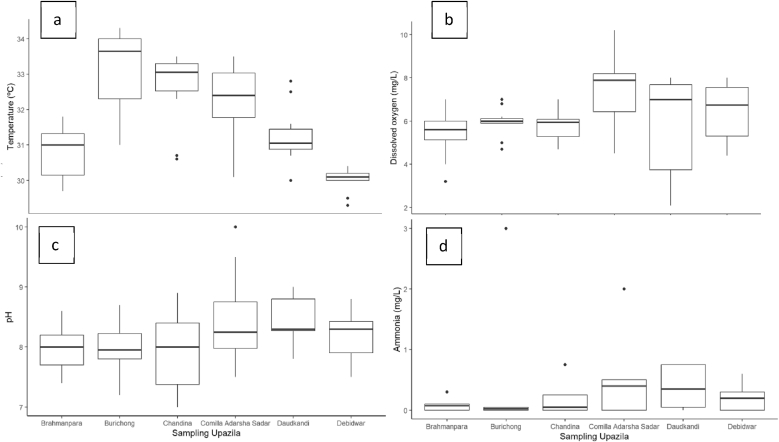
Water quality parameters (a) Temperature, (b) Dissolved Oxygen, (c) pH, and (d) Ammonia across six sampling Upazila of Cumilla District.

### 3.3 Colony forming unit (CFU)

**Fig. 4:**
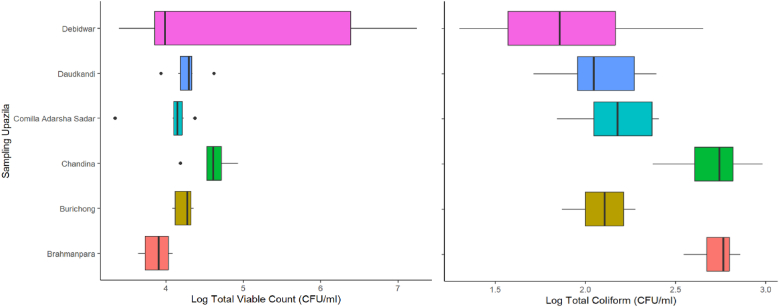
Total plate count and total coliform count (CFU/ml) of aquaculture water collected from Cumilla Districts.

The total bacterial load, as indicated by total plate count (TPC), and the presence of total coliforms varied significantly across different sampling locations in the Cumilla District. The data reflects spatial variation in microbial contamination across the aquaculture systems in different Upazilas, while some areas (Chandina and Debidwar) show significantly higher levels of both total bacteria (2.48 × 10^3^ to 1.75 × 10^7^ CFU/ml) and coliforms (2.0 × 10^1^ to 9.59 × 10^2^ CFU/ml). Therefore, we can conclude that the Debidwar as the most contaminated site, both in terms of total bacterial load and coliforms, whereas Brahmanpara consistently showed the lowest total (1.10 × 10^4^ CFU/ml) contamination but higher in (7.20 × 10^2^ CFU/ml) total coliform count (Fig. 4). This discrepancy may point to specific fecal contamination sources or inadequate sanitary practices despite relatively low overall bacterial density [11]. These findings highlight the need for improved water management, hygiene practices, and routine microbial monitoring to ensure the safety and productivity of aquaculture environments [29].

### 3.4 Morphological characteristics of isolates

A total of 35 bacterial isolates were obtained from freshwater aquaculture pond raw samples using selective agar media, specifically Eosin Methylene Blue (EMB) agar. Bacterial isolates were selected based on morphological characteristics of these isolates on EMB media which is given in Table S5. Most of the colonies were circular where a few irregular or rhizoid shaped colonies were also observed (e.g., ComW4C1 and BurW5C1). This is because of the possible variation in growth behavior or motility (Ozcan 2021). Colonies were small to large, and margins were predominantly entire. In some isolates undulate or lobate edges were observed [30]. Elevation of the colonies were flat, raised or convex (e.g., DebW6C1 and DebW6C3), and textures were generally smooth, with rough or slightly rough colonies which indicated older or more resilient bacterial strains.

### 3.5 Characteristics on EMB agar

In EMB agar, bacterial colonies exhibited a characteristic green or violet metallic sheen which are typical indicators of strong lactose fermentation and indicating the presence of *Escherichia coli* [30]. In contrast, bacterial isolates developed pink, purple, or pinkish-violet colonies without any metallic sheen. The absence of a metallic sheen but the presence of pigmentation suggests moderate lactose fermentation, and presence of *Enterobacter* spp., *Klebsiella* spp., or *Citrobacter* spp. [31]. These bacteria are commonly found in aquatic environments and are known to contribute to water quality degradation when present in excess. Bacterial isolates produced creamy, off-white, or white colonies, indicating an absence of lactose fermentation and belonging to non-lactose Gram-negative bacteria such as *Salmonella* spp., *Shigella* spp., or *Proteus* spp. These organisms often associated with pathogenicity in aquatic or clinical environments [24]. Additionally, some isolates developed purple or deep violet colonies. These may represent either weak lactose fermenters or atypical strains of *E. coli* that do not produce the classic metallic sheen under the given culture conditions. In summary, based on colony characteristics on EMB agar, approximately 20–25 % of the isolates are likely *E. coli*, showing strong lactose fermentation with a metallic sheen. Around 50–60 % are presumptive other coliforms such as *Enterobacter* and *Klebsiella*, while 15–20 % may be non-lactose fermenters like *Salmonella* or *Shigella*. While these interpretations offer useful preliminary identification, confirmation through biochemical tests or molecular techniques (e.g., PCR or 16S rRNA gene sequencing) is essential for precise species identification [32].

### 3.6 Characteristics on MacConkey agar

In MacConkey agar, Lactose-fermenting bacteria such as bacterial isolates produce pink to purple colonies. The pink coloration arises due to acid production from lactose fermentation, which lowers the pH and activates the neutral red pH indicator in the medium [24]. These results suggest the presence of potential coliforms such as *Escherichia coli, Klebsiella* spp., and other Enterobacteriaceae. On the other hand, colorless colonies are indicative of non-lactose fermenting gram-negative bacteria, possibly including species such as *Salmonella, Shigella*, or *Pseudomonas*. These organisms do not ferment lactose, and hence, the colonies do not change color on the medium. Purple colonies observed suggest strong lactose fermenters or high acid producers, often associated with fecal contamination. Interestingly, no bacterial growth was recorded in ChaW1C2, ChaW2C1, and ChaW3C1, which may be due to the absence of gram-negative bacteria in those samples or inhibitory effects of bile salts and crystal violet on sensitive strains [30]. Overall, the colony color variations across different upazilas suggest heterogeneous bacterial populations in the sampled water sources, with varying degrees of fecal contamination and presence of both lactose-fermenting and non-fermenting gram-negative bacteria. These preliminary phenotypic results support further molecular confirmation and antibiotic resistance profiling.

### 3.7 Antibiotic sensitivity and multi-drug resistance index (MDRI) of bacterial isolates

#### 3.7.1 Antibiotic sensitivity

**Fig. 5:**
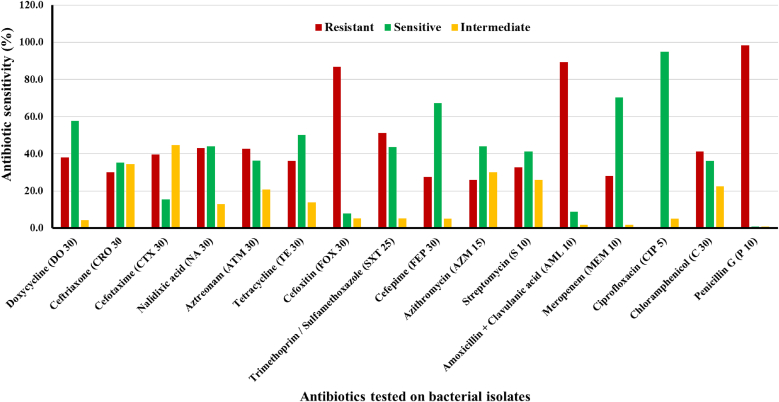
Antibiotic sensitivity of Bacterial isolates isolated from aquaculture water of Cumilla District.

Antibiotic sensitivity results showed extensive resistance among isolates, particularly against third-generation cephalosporins like cefotaxime (CTX) and ceftriaxone (CRO), as well as aztreonam (ATM), cefoxitin (FOX), and tetracycline (TE). Isolates from Debidwar, Comilla Adarsha Sadar, and Daudkandi were particularly resistant, with multiple strains displaying resistance (R) across more than 10 antibiotics, especially β-lactams and macrolides. In contrast, some isolates from Chandina and Burichong exhibited comparatively higher susceptibility, particularly to Ciprofloxacin, Sulphamethoxazole, Meropenem, and Chloramphenicol (Fig. 5). Among the antibiotics tested, Meropenem (MEM 10) exhibited the highest efficacy, with approximately 90 % of isolates showing sensitivity. Ciprofloxacin (CIP 5) also demonstrated strong antibacterial activity, with about 80 % sensitivity. In contrast, Chloramphenicol (C 30) showed only around 40 % sensitivity, indicating moderate effectiveness against coliform bacteria in the aquaculture environment [33]. The relatively low resistance to Meropenem suggests that carbapenem resistance has not yet become widespread among these isolates.

Moderate levels of sensitivity were observed for Doxycycline (DO 30) and Trimethoprim/Sulphamethoxazole (SXT 25), where about 50–65 % of isolates responded favorably. Antibiotics such as Azithromycin (AZM 15), Cefepime (FEP 30), and Streptomycin (S 10) also displayed intermediate effectiveness, with noticeable proportions of sensitive, resistant, and intermediate responses (Fig. 5). Although these antibiotics still have limited therapeutic use, careful use is advised for their inconsistent performance. On the other hand, bacterial isolates showed high resistance against different β-lactam antibiotics. For example, Penicillin-G (P 10) exhibited almost 100 % resistance against different bacterial isolates which means this antibiotic is ineffective in treating these isolates. Another antibiotic named Cefoxitin (FOX 30) was nearly 85 % resistant against the isolates which suggested the presence of ESBL or AMP C β-lactamase-producing bacteria. Different third-generation cephalosporins like Cefotaxime (CTX 30), Ceftriaxone (CRO 30) and Aztreonam (ATM 30) also showed significant resistance further proving the presence of ESBL producing bacteria. Besides, Amoxycillin in combination with Clavulanic acid (AMC/AML) which is usually applied against β-lactamase-producing bacteria exhibited high resistance (approximately 80 %) against isolated bacterial strains. This condition indicates presence of potential inhibitor-resistant strains. Substantial intermediate resistance has been observed in case of Azithromycin (AZM 15), Tetracycline (TE 30) and Nalidixic acid (NA 30) which indicated that antibiotic resistance has been partially expressed or emerging. The risk of ESBL-producing coliforms is highlighted by the presence of resistant to critically resistant antibiotics particularly cephalosporins in aquatic environments. The present condition emphasizes the necessity of stringent antibiotic stewardship and environmental monitoring [34].

#### 3.7.2 Multi drug resistance index (MDRI)

**Fig. 6:**
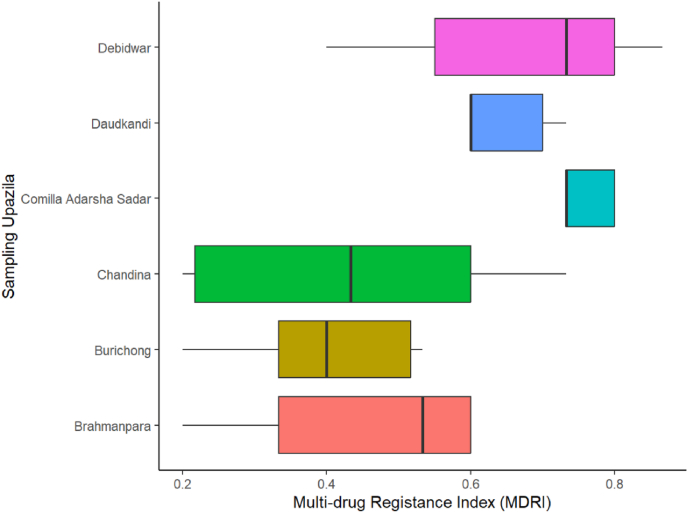
Multi-drug resistance index (MDRI) of bacterial isolates isolated from aquaculture water of Cumilla Districts.

In different sampling sites, the MDRI values varied differently indicating varying degrees of resistance among the isolates. The value ranged from as low as 0.19 to as high as 0.81 (Fig. 6). This high MDRI value is observed in the bacterial isolates of Comilla Adarsha Sadar (0.75), Debidwar (0.81) and Daudkandi upazila (0.69). The higher MDRI values in the above mentioned upazilas indicate intensive antibiotic exposure or contamination in the aquaculture environments (Tambekar et al., 2006). In contrast, a wide spread of MDRI values were observed in Chandina, ranging from as low as approximately 0.19–0.69, reflecting variability in resistance patterns within this upazila, possibly due to inconsistent antibiotic practices among different farms [35]. Similarly, Brahmanpara also showed moderate MDRI levels where Burichong had relatively lower MDRI values. These values demonstrate minimal exposure compared to other higher values observed in other sampling upazila. However, MDRI values in studied areas are considerably higher than the threshold value 0.2. The threshold value of 0.2, which is typically considered the benchmark for identifying high-risk environments regarding antimicrobial resistance [36]. The MDRI values of different locations of Cumilla District indicate an alarming level of multi-drug resistance among coliform bacteria in aquaculture systems.

#### 3.7.3 Extended-spectrum beta-lactamase (ESBL)-producing coliform bacteria

**Fig. 7:**
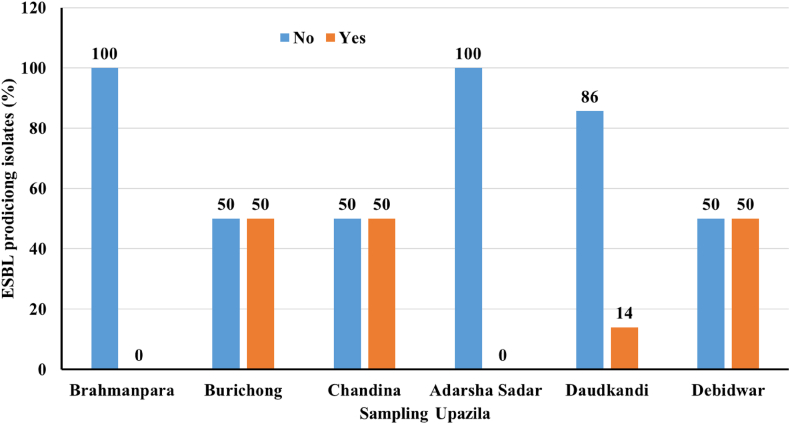
Prevalence of extended-spectrum beta-lactamase (ESBL)-producing coliform bacteria from aquaculture pond water of Cumilla District.

The distribution of ESBL-producing coliform bacterial isolates across six upazilas of Cumilla District are represented in graph 7 (Fig. 7). Significant spatial variation was seen in the prevalence of ESBL producers. Burichong, Chandina, and Debidwar each exhibited a 50 % ESBL positivity rate which reflected a concerning presence of ESBL-producing coliforms in these aquaculture environments. In contrast, in case of Brahmanpara and Comilla Adarsha Sadar, none of the isolates tested positive for ESBL production. Daudkandi showed the lowest ESBL positivity among the upazilas with only 14 % of isolates testing positive (Fig. 7).

These findings highlight the uneven distribution of ESBL-producing bacteria across the region with certain upazilas such as Burichong, Chandina, and Debidwar emerging as potential hotspots for antimicrobial resistance. The presence of ESBL-producing coliforms in aquaculture environments is a serious public health concern, as it emphasizes the potential for horizontal gene transfer of resistance traits and the contamination of aquatic food products with multidrug-resistant pathogens [37]; [31]. This distribution underscores the importance of localized surveillance and targeted interventions to mitigate the spread of resistant pathogens in aquaculture systems ([38]; [39]; [40]).

#### 3.7.4 Prevalence of antibiotic-resistant genes

**Fig. 8:**
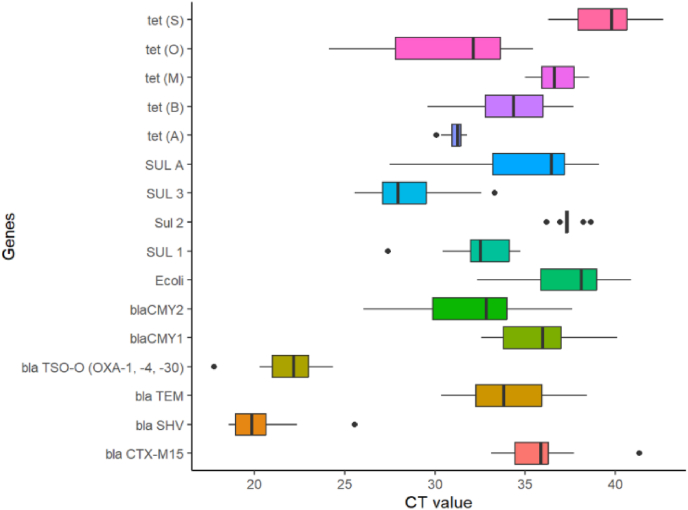
Prevalence of resistance genes in the bacterial isolates isolated from Cumilla Districts aquaculture pond waters.

A wide range of antibiotic resistance genes (ARGs) were identified during quantitative PCR (qPCR) analysis of aquaculture water samples from different sites of Cumilla District. These included genes showing resistance to tetracyclines (tetA, tetB, tetM, tetS), sulfonamides (sul1, sul2, sul3, sulA) and β-lactams (CMY-1, CMY-2, CTX-M15, SHV, TEM). Also, some mobile genetic elements (TSO) were also detected (Fig. 8). The blaSHV, blaTEM, and blaTSO-O were detected at the lowest cycle threshold (CT) values (e.g., SHV: 18.29 in DebW6C2) among all these genes which reflecting high gene abundance and a significant prevalence of ESBL-producing bacteria in the aquatic environment. These findings are consistent with Paterson and Bonomo [39], who reported SHV-type β-lactamases as major contributors to ESBL resistance in both clinical and environmental sources.

Moderate CT values (approximately 25–30) for CMY-1/2, sul2, and sulA suggest the presence of AmpC β-lactamase producers and sulfonamide-resistant bacteria, potentially due to the common use of sulfonamides in aquaculture. This mirrors previous observations by Jacoby [40] and Alm et al. [41], who linked such resistance to environmental contamination from agricultural and aquacultural runoff. Although CTX-M15 was detected at higher CT values (up to ∼41), its clinical relevance remains a concern, as it confers resistance to third-generation cephalosporins, a pattern also documented by Ma et al. [42] and Xiong et al. [43] in aquaculture systems. Tetracycline resistance genes were detected but with generally higher CT values (>30), suggesting lower gene abundance [44]. However, the mobile genetic element TSO was consistently observed at low CT values (e.g., 21.79 in BurW1C1), indicating a high potential for horizontal gene transfer. This highlights the risk of ARGs dissemination in aquaculture environments. Overall, the predominance of β-lactam and sulfonamide resistance genes reflects the role of aquaculture water as a reservoir of ARGs, reinforcing the need for antibiotic stewardship and environmental surveillance [45].

## 4 Conclusion

This study inspected the aquaculture system in east-south part of Bangladesh, mainly Cumilla District for the presence of ESBL-producing coliform bacteria and their antibiotic resistance pattern. The physicochemical parameters of pond water were poor in some of the sampling regions pond waters such as high ammonia and low DO. Total viable count and total coliform count indicated a substantial unacceptable limit of bacterial colony in the aquaculture water of studied samples. Antibiotic susceptibility tests revealed that all isolates were resistant to multiple tested antibiotics, with resistance to commonly used antibiotics like Amoxycillin, Penicillin G, and cephalosporins. Meanwhile, Ciprofloxacin and Meropenem showed better effectiveness. The MDR Index values were all above 0.2, indicating a high-risk environment. Around one-third of the isolates were found to be ESBL-producers, and molecular tests confirmed the presence of important resistance genes such as bla SHV, bla TEM, Sul1, and Sul2. Though in our results we did not find any strong correlation between total bacterial count and antibiotic resistance pattern. But the prevalence of coliform bacteria and higher resistance scenario are supported by the pond water quality and management techniques of these regions. These findings highlight that aquaculture water in the study area can act as a reservoir for drug-resistant bacteria, which may pose a risk to aquatic life and public health. Proper management, monitoring, and responsible use of antibiotics in aquaculture are essential to limit the spread of antimicrobial resistance. However, more detailed research based on high thought metagenomics needs to be incorporated for better understanding of AMR picture in aquaculture industry.

## CRediT authorship contribution statement

**Rakibul Islam:** Writing – original draft, Methodology, Investigation. **Nazia Afrin:** Writing – review & editing, Writing – original draft, Methodology. **Md Shakhawate Hossain:** Writing – review & editing, Writing – original draft, Visualization, Validation, Supervision, Resources, Methodology, Funding acquisition, Formal analysis, Data curation, Conceptualization.

## Ethics approval and consent to participate

The study did not involve any endangered or protected species. No specific permission was required for the locations and activities involved in this study. All experimental manipulations were conducted following the principles of the Ethical Committee of Gazipur Agricultural University, Bangladesh, and the National Research Ethics Committee (NREC) of Bangladesh.

## Data availability statement

The data is available on request.

## Declaration of competing interest

The authors declare that they have no known competing financial interests or personal relationships that could have appeared to influence the work reported in this paper.
