# Supplementary material for: Unveiling the presence of ESBL-producing coliform bacteria in the aquaculture system of Cumilla District of Bangladesh
Source: New Microbes New Infect. 2026 Jan 16;70:101706. doi: 10.1016/j.nmni.2026.101706 (PMC12857353; doi:10.1016/j.nmni.2026.101706)
Supplement: Multimedia component 1 [file mmc1.docx]

**Unveiling the Presence of ESBL-Producing Coliform Bacteria in the Aquaculture System of Cumilla District of Bangladesh**

Rakibul Islam^1^, Nazia Afrin^2^, Md Shakhawate Hossain^1,^ *

^1^ Department of Fisheries Biology and Aquatic Environment, Gazipur Agricultural University, Gazipur 1706, Bangladesh

**^2^**Laboratory of Microbiology, Department of Botany, Jahangirnagar University, Savar 1342, Bangladesh

*Corresponding author

Md Shakhawate Hossain, PhD ([shakhawate@gau.edu.bd](mailto:shakhawate@gau.edu.bd))

Telephone: +8801884372094

**Table S1: Sampling locations with their GPS coordinate**

| **District** | **Upazila** | **Pond No.** | **Latitude** | **Longitude** |
| --- | --- | --- | --- | --- |
| Comilla | Comilla Adarsha Sadar | Com 1 | 23.464087 | 91.230073 |
| Comilla | Comilla Adarsha Sadar | Com 2 | 23.463332 | 91.229635 |
| Comilla | Comilla Adarsha Sadar | Com 3 | 23.463432 | 91.233787 |
| Comilla | Comilla Adarsha Sadar | Com 4 | 23.463255 | 91.234428 |
| Comilla | Comilla Adarsha Sadar | Com 5 | 23.455968 | 91.235252 |
| Comilla | Comilla Adarsha Sadar | Com 6 | 23.448535 | 91.234567 |
| Comilla | Chandina | Cha 1 | 23.468412 | 90.941568 |
| Comilla | Chandina | Cha 2 | 23.467625 | 90.941763 |
| Comilla | Chandina | Cha 3 | 23.453814 | 90.9397 |
| Comilla | Chandina | Cha 4 | 23.455238 | 90.972982 |
| Comilla | Chandina | Cha 5 | 23.456058 | 90.973052 |
| Comilla | Chandina | Cha 6 | 23.45956 | 90.969493 |
| Comilla | Debidwar | Deb 1 | 23.584012 | 91.01067 |
| Comilla | Debidwar | Deb 2 | 23.584053 | 91.010145 |
| Comilla | Debidwar | Deb 3 | 23.583093 | 91.102095 |
| Comilla | Debidwar | Deb 4 | 23.54858 | 91.016003 |
| Comilla | Debidwar | Deb 5 | 23.548333 | 91.015673 |
| Comilla | Debidwar | Deb 6 | 23.546975 | 91.016415 |
| Comilla | Burichong | Bur 1 | 23.551482 | 91.139713 |
| Comilla | Burichong | Bur 2 | 23.559892 | 91.100457 |
| Comilla | Burichong | Bur 3 | 23.558808 | 91.14742 |
| Comilla | Burichong | Bur 4 | 23.551264 | 91.148209 |
| Comilla | Burichong | Bur 5 | 23.552012 | 91.09088 |
| Comilla | Burichong | Bur 6 | 23.483214 | 91.056321 |
| Comilla | Brahmanpara | BP 1 | 23.602008 | 91.106662 |
| Comilla | Brahmanpara | BP 2 | 23.603098 | 91.103308 |
| Comilla | Brahmanpara | BP 3 | 23.60166 | 91.10333 |
| Comilla | Brahmanpara | BP 4 | 23.601675 | 91.10249 |
| Comilla | Brahmanpara | BP 5 | 23.600655 | 91.102045 |
| Comilla | Brahmanpara | BP 6 | 23.601325 | 91.11018 |
| Comilla | Daudkandi | DK 1 | 23.530795 | 90.84574 |
| Comilla | Daudkandi | DK 2 | 23.530487 | 90.846022 |
| Comilla | Daudkandi | DK 3 | 23.529497 | 90.815908 |
| Comilla | Daudkandi | DK 4 | 23.528328 | 90.816187 |
| Comilla | Daudkandi | DK 5 | 23.531483 | 90.815985 |
| Comilla | Daudkandi | DK 6 | 23.532383 | 90.816043 |

**Table S2: Antibiotic Susceptibility Testing Breakpoints Table (ECAS, 2024)**

| **Sl No.** | **Antibiotic Name** | **Resistant** | **Intermediate** | **Sensitive** |
| --- | --- | --- | --- | --- |
| 1 | Aztreonam (**ATM 30**) | ≤21 | 22-25 | ≥26 |
| 2 | Cefotaxime (**CTX 30**) | ≤14 | 15-22 | ≥23 |
| 3 | Amoxicillin + Clavulanic acid (**AML 10**) | ≤19 | - | ≥20 |
| 4 | Cefepime (**FEP 30**) | ≤14 | 15-17 | ≥18 |
| 5 | Azithromycin (**AZM 15**) | ≤13 | 14−17 | ≥18 |
| 6 | Penicillin G (**P 10**) | ≤19 | 20-27 | ≥28 |
| 7 | Chloramphenicol (**C 30**) | ≤12 | 13-17 | ≥18 |
| 8 | Ceftriaxone (**CRO 30**) | ≤13 | 14-20 | ≥21 |
| 9 | Trimethoprim / Sulfamethoxazole (**SXT 25**) | ≤14 | 15-16 | ≥17 |
| 10 | Ciprofloxacin (**CIP 5**) | ≤15 | 16-20 | ≥21 |
| 11 | Tetracycline (**TE 30**) | ≤11 | 12−14 | ≥15 |
| 12 | Meropenem (**MEM 10**) | ≤13 | 14-15 | ≥16 |
| 13 | Cefoxitin (**FOX 30**) | ≤10 | 11-12 | ≥13 |
| 14 | Nalidixic acid (**NA 30**) | ≤13 | 14-18 | ≥19 |
| 15 | Doxycycline (**DO 30**) | ≤10 | 11−13 | ≥14 |
| 16 | Streptomycin (**S 10**) | ≤11 | 12−14 | ≥15 |

**Table S3:** Primers used for qRT-PCR analysis.

| **Name of Genes** | **Sequence 5'-3'** | **Name of Genes** | **Sequence 5'-3'** |
| --- | --- | --- | --- |
|  |  |  |  |
| ***bla*** ***_CTX-M15_*** | F- GTGATACCACTTCACCTC  R-AGTAAGTGACCAGAATCAG | *sul*A | F- TCTTGAGCAAGCACTCCAGCAG  R- TCCAGCCTTAGCAACCACATGG |
|  |  |  |  |
| ***bla*** ***_SHV_*** | F-ACTATCGCCAGCAGGATC  R-ATCGTCCACCATCCACTG | *tet* (A) | F- GTGAAACCCAACATACCCC  R- GAAGGCAAGCAGGATGTAG |
|  |  |  |  |
| ***bla*** ***_TEM_*** | F- GATCTCAACAGCGGTAAG  R- CAGTGAGGCACCTATCTC | *tet* (B) | F- CCTTATCATGCCAGTCTTGC  R- ACTGCCGTTTTTTCGCC |
|  |  |  |  |
| ***bla*** ***_TSO-O (OXA-1_*_,_*_-4_*_,_*_-30)_*** | F- GGCACCAGATTCAACTTTCAAG  R- GACCCCAAGTTTCCTGTAAGTG | *tet* (M) | F- GTG GAC AAA GGT ACA ACG AG  R- CGG TAA AGT TCG TCA CAC AC |
|  |  |  |  |
| *bla*CMY1 | F- TGAAACTGGGCTATTTGAACGC  R- TTGGTGGGTCAGGGAGATGG | *tet* (O) | F- AAC TTA GGC ATT CTG GCT CAC  R - TCC CAC TGT TCC ATA TCG TCA |
|  |  |  |  |
| *bla*CMY2 | F- ACAGCCTCTTTCTCCACATTTG  R- GCCAGTTCAGCATCTCCCAG | *tet* (S) | F- CAT AGA CAA GCC GTT GAC C  R - ATG TTT TTG GAA CGC CAG AG |
|  |  |  |  |
| *sul*1 | F- CGCACCGGAAACATCGCTGCAC  R- TGAAGTTCCGCCGCAAGGCTCG | *Sul*3 | F- TCCGTTCAGCGAATTGGTGCAG  R- TTCGTTCACGCCTTACACCAGC |
|  |  |  |  |
| *Sul*2 | F- TCCGGTGGAGGCCGGTATCTGG  R- CGGGAATGCCATCTGCCTTGAG |  |  |

**Table S4: Observed water quality parameters of different aquaculture ponds in Cumilla District and comparison with standard reference values**

| **Sample Upazila** | **pH** | **DO (mg/L)** | **Temperature (ºC)** | **Ammonia (mg/L)** |
| --- | --- | --- | --- | --- |
| Comilla Adarsha Nagar | 7.9 - 10 | 4.5 – 10.2 | 30.3 – 33.5 | 0 – 2.0 |
| Chandina | 7.7- 8.9 | 4.7 – 6.8 | 30.6 – 33.5 | 0 - 0.75 |
| Debidwar | 7.8- 8.4 | 4.4 – 7.7 | 29.3 – 30.2 | 0 - 0.6 |
| Burichong | 7.8- 8.3 | 4.7 – 6.8 | 31.3 - 34.3 | 0 – 3.0 |
| Brahmapara | 7.7- 8.4 | 3.2 – 6.7 | 29.7 – 31.8 | 0 - 0.3 |
| Daudkandi | 7.8- 9 | 2.1- 7.9 | 30.8- 32.8 | 0 – 0.75 |
| **Total Range** | **7.7 – 10.0** | **2.1 – 10.2** | **29.3 – 34.3** | **0 – 3.0** |
| Optimum range for aquaculture | 6.5 – 8.5 (Boyd, 1998; Global Seafood Alliance, 2017) | >5.0 mg/L (Boyd, 1998) | 26°C – 32°C (Halim et al., 2018) | <0.05 mg/L (University of Florida IFAS Extension, 2023) |
| Remarks | Mostly within acceptable range; some samples (e.g., Com 6) exceeded optimal | Some critical low values (e.g., DK 1, DK 2, BP 1) indicating potential stress | Generally acceptable, though higher temperatures (>34°C) at Bur 3 | Several high values (e.g., Bur 3, Com 6) indicating potential toxicity |

**Table S5. List of the bacterial colony grows in selective agar media and their characteristics**

| **Sample ID** | **Isolates** | **Characteristics of bacterial colony** | | | | | | | |
| --- | --- | --- | --- | --- | --- | --- | --- | --- | --- |
|  |  | **Shape** | **Size** | **Margin** | **Elevation** | **Texture** | **Appearance** | **Pigmentation** | **Optical Property** |
| DebW1 | DebW1C1 | Circular | Small | Entire | Raised | Smooth | Glistening | Creamy | Translucent |
|  | DebW1C2 | Circular | Medium | Entire | Raised | Smooth | Glistening | Creamy Yellow | Opaque |
| DebW2 | DebW2C1 | Circular | Medium | Entire | Convex | Smooth | Glistening | Pinkish Violet | Opaque |
|  | DebW2C2 | Circular | Large | Entire | Raised | Rough | Rough | Pink | Opaque |
| DebW3 | DebW3C1 | Circular | Large | Entire | Raised | Rough | Glistening | Violet | Opaque |
|  | DebW3C2 | Circular | Medium | Entire | Convex | Smooth | Glistening | Deep Violet | Opaque |
| DebW6 | DebW6C1 | Oval shape | Small | Entire | Flat | Smooth | Glistening | Violet (Metal sheen) | Opaque |
|  | DebW6C2 | Circular | Large | Entire | Convex | Smooth | Glistening | Off white | Opaque |
|  | DebW6C3 | Circular | Large | Entire | Flat | Smooth | Glistening | Pink | Opaque |
|  | DebW6C4 | Circular | Medium | Entire | Raised | Smooth | Glistening | Baby Pink | Opaque |
| ComW1 | ComW1C1 | Circular | Large | Entire | Raised | Rough | Glistening | Purple | Opaque |
| ComW3 | ComW3C1 | Circular | Large | Entire | Convex | Smooth | Glistening | Violet | Opaque |
| ComW4 | ComW4C1 | Ryzoid | Large | Lobate | Raised | Smooth | Glistening | Violet | Opaque |
|  | ComW4C2 | Circular | Large | Entire | Convex | Smooth | Glistening | Violet | Opaque |
| ComW6 | ComW6C1 | Circular | Medium | Entire | Raised | Smooth | Glistening | Pinkish Violet | Opaque |
| ChaW1 | ChaW1C1 | Circular | Small | Entire | Raised | Smooth | Glistening | Pink | Opaque |
|  | ChaW1C2 | Circular | Large | Entire | Raised | Rough (less) | Rough | Off white | Opaque |
| ChaW2 | ChaW2C1 | Circular | Medium | Entire | Raised | Smooth | Glistening | Pinkish Violet | Opaque |
| ChaW3 | ChaW3C1 | Circular | Medium | Entire | Convex | Rough | Rough | Pink | Opaque |
| ChaW4 | ChaW4C1 | Irregular | Large | Undulate | Raised | Smooth | Glistening | Light Pink | Opaque |
| ChaW6 | ChaW6C1 | Circular | Small | Entire | Raised, Spreading edge | Smooth | Glistening | Pinkish Violet | Opaque |
| DKW1 | DKW1C1 | Irregular | Medium | Undulate | Raised | Smooth | Glistening | Off white | Opaque |
|  | DKW1C2 | Circular | Medium | Entire | Convex | Rough | Rough | Pink | Opaque |
| DKW2 | DKW2C1 | Circular | Small | Entire | Convex | Smooth | Glistening | Violet | Opaque |
| DKW4 | DKW4C1 | Circular | Large | Entire | Raised | Rough | Rough | Pink | Opaque |
|  | DKW4C2 | Circular | Medium | Entire | Raised | Rough | Rough | Pink | Opaque |
| DKW6 | DKW6C1 | Circular | Medium | Entire | Convex | Smooth | Glistening | Pinkish white | Opaque |
|  | DKW6C2 | Circular | Small | Entire | Convex | Smooth | Glistening | White | Opaque |
| BurW1 | BurW1C1 | Circular | Medium | Entire | Convex | Rough | Rough | Pink | Opaque |
| BurW2 | BurW2C1 | Circular | Small | Entire | Convex | Smooth | Glistening | Light Pink | Opaque |
| BurW3 | BurW3C1 | Circular | Small | Entire | Convex | Smooth | Glistening | Violet (metal sheen) | Opaque |
| BurW4 | BurW4C1 | Circular | Small | Entire | Convex | Smooth | Glistening | Violet (metal sheen) | Opaque |
| BurW5 | BurW5C1 | Irregular | Small | Undulate | Convex | Smooth | Glistening | Pink (metal sheen) | Opaque |
| BurW6 | BurW6C1 | Circular | Small | Entire | Convex | Smooth | Glistening | Violet (metal sheen) | Opaque |
| BPW2 | BPW2C1 | Circular | Medium | Entire | Raised | Smooth | Glistening | Light Pink (Green metal sheen) | Opaque |


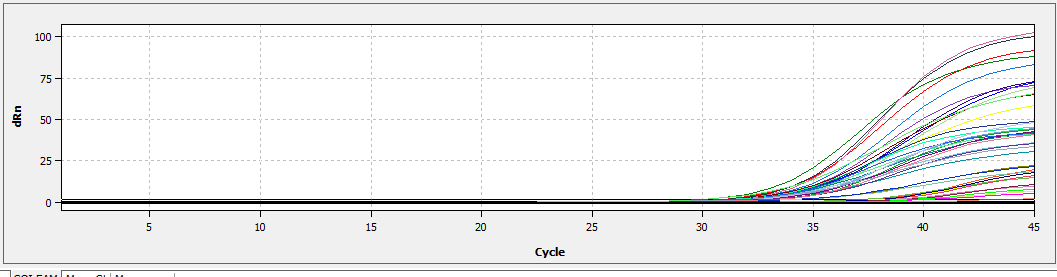

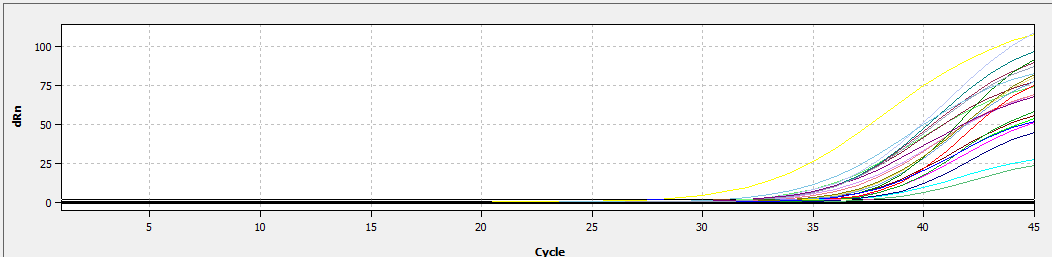

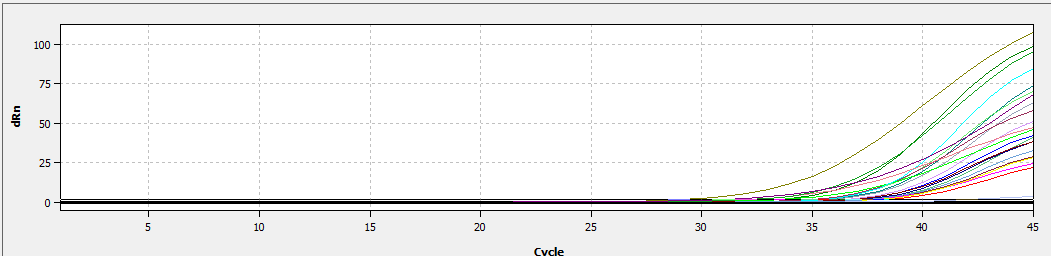

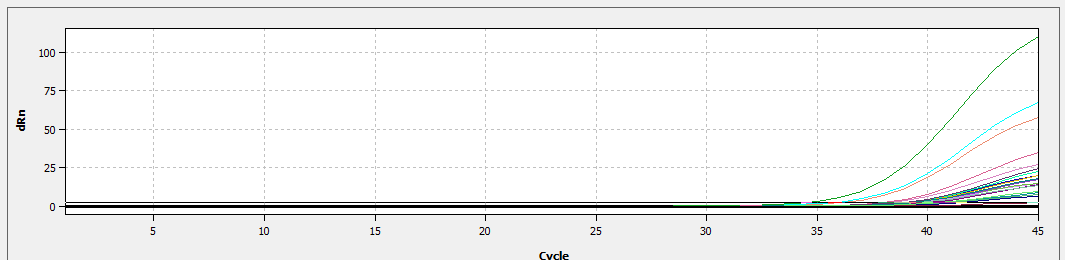


**Fig S1.** Representative Real-time qRT-PCR amplification curves of target gene expression.
